# Towards Sustainable Personalized Assembly Through Human-Centric Digital Twins

**DOI:** 10.3390/s25185662

**Published:** 2025-09-11

**Authors:** Marina Crnjac Zizic, Nikola Gjeldum, Marko Mladineo, Bozenko Bilic, Amanda Aljinovic Mestrovic

**Affiliations:** Faculty of Electrical Engineering, Mechanical Engineering and Naval Architecture, University of Split, R. Boskovica 32, 21000 Split, Croatia; ngjeldum@fesb.hr (N.G.); mmladine@fesb.hr (M.M.); bbilic@fesb.hr (B.B.); amanda.aljinovic@fesb.hr (A.A.M.)

**Keywords:** personalized production, Industry 4.0/5.0, ExCurS project, human-centric approach, digital twin, decision-making

## Abstract

New trends in industry emphasize green and sustainable production on the one hand and personalized or individualized production on the other hand. Introducing new manufacturing technologies and materials to integrate the customer’s specific requirements into the product, while keeping the focus on environmental footprint, becomes a serious challenge. As a result, new production paradigms are developed to keep up with new trends. The most known Industry 4.0 paradigm is oriented towards new technologies and digitalization. Recently, Industry 5.0 appeared as a supplement to the existing Industry 4.0 paradigm, oriented to sustainability and the worker. A multidisciplinary approach is necessary to address these challenges. The Industry 5.0 paradigm’s main pillars—human centricity, resilience, and sustainability—are also pillars of the multidisciplinary approach used in this research. A human-centric approach includes workforce reskilling and acquiring new technologies to ensure that technology serves to enhance human work, while creating a supportive and inclusive work environment and prioritizing employee engagement and wellbeing. Resilience as a second pillar is related to the ability of manufacturing systems and processes to adapt to changing conditions to remain robust and flexible, and sustainability is an important and long-term requirement of this multidisciplinary approach. Based on the research part of the Erasmus+ ExCurS project, particularly research focused on application and training related to digital twins, an advanced concept of organizational sustainability is presented in this paper. The concept of organizational sustainability is realized through the usage of key digital twin technologies aligned with human-centric approaches. A new prototype of a digital twin that optimizes an assembly system based on a developed algorithm and humanoid decision-making is provided as a proof of concept. The human-centric digital twin for industrial application is presented through a case study of personalized products.

## 1. Introduction

### 1.1. Challenges of Personalized Production

**Figure 1:**
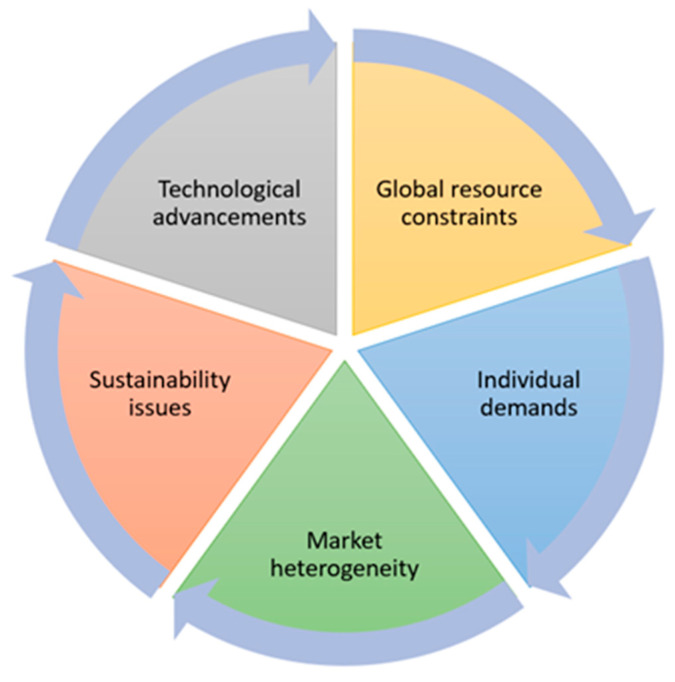
Paradigm shift driven by key areas.

Today, increasing market heterogeneity and the long-term goals of sustainability are causing a paradigm shift in manufacturing industry. This paradigm shift is enabled by emerging information and communication technologies [1] such as cyber-physical systems (CPSs), Internet of Things (IoT), and cloud computing. Additionally, there is growing human awareness about sustainability. Popular discussion regarding “Global drivers” such as population, demographics, food security, energy security, and community security emphasizes the responsibility of the manufacturing sector to contribute substantially to the role of humans in industry regarding complex decision-making and ethical considerations (especially safety and potential risks) while ensuring that technology aligns with broader societal goals [2] and ecological goals [3]. Therefore, Industry 5.0 [4] appears as supplement to the existing Industry 4.0 paradigm [5] with a focus on the worker, whose role is nevertheless irreplaceable in the production process [6]. There are also scientific projects, like the ExCurS project on which this research is based [3], that are trying to address the skills of human workers, new digital technologies, and sustainability challenges at the same time. Furthermore, there are various terms and definitions of Industry 5.0 in the literature, which help to deepen the understanding of Industry 5.0 from different perspectives [7]. Several enabling technological trends [8] such as Internet of Things (IoT) [9], digital twin (DT) [10], big data analytics [11], cobots [12], 6G, and blockchain (BC) [13] are integrated with cognitive skills and innovation that can help industries increase production and deliver customized products more quickly [14]. This transformation explains how manufacturing systems respond better to key areas in paradigm shift (Figure 1), enabling more efficient, flexible, and personalized production processes [15]. However, it is not sufficient to only list the enabling technologies; their true importance lies in how they are integrated to support personalized production. The Internet of Things (IoT), for example, provides real-time data from machines and processes, which is then used by digital twins for accurate simulations and the optimization of individualized products. Big data analytics supports this by identifying customer-specific patterns, cobots ensure flexible collaboration with human workers, and blockchain guarantees trust and traceability in personalized value chains. By positioning digital twins as the central integrating framework, supported by IoT, data analytics, cobots, and blockchain, the link between these technologies and the goals of Industry 5.0 in personalized production becomes clearer.

Moreover, the trend towards assembly customization and batch size production in different industries leads to ever-changing product configurations and therefore generates a unique data history for each product. The complexity of the product is further enhanced by the continuous removal of exclusively mechanical components and the ever-increasing proportion of mechatronic parts, which consist of mechanical, electrical, and software elements [16]. Mechatronic products often consist of thousands of different and heterogeneous parts and therefore require a wide range of manufacturing technologies. Such products are assembled in increasingly decentralized production processes, often in geographically and organizationally distributed supply chains [17].

Many manufacturing companies today use information technology (IT) systems that are heterogeneous, difficult, and expensive to improve and maintain. To solve this problem there are numerous data-driven approaches [18] that enable interoperability between IT systems [19]. However, these integration solutions are not flexible enough to quickly adapt to changes in the company’s production processes [20]. Among them, the integration of the Business Process Management Notation (BPMN) model [21] and blockchain technology appears as a compelling solution, promising a revolution in assembly process management. Although blockchain has been primarily applied in financial applications, solutions based on blockchain (BC) technologies are gaining more and more attention in the context of new industrial paradigms [22]. The concept of a digital twin is almost twenty years old, posing an enhancement of well-known concepts like Computer-Integrated Manufacturing (CIM) and Product Lifecycle Management (PLM), with expected benefits through ubiquitous simulation, real-time analysis, and synchronous processing [23]. Digital twin has become a key word in engineering, society, and medicine, and it is also a popular topic in research aimed at creating virtual data-driven replicas of real objects and simulating their behaviors to predict and optimize the entire system’s functioning [24]. DTs can mirror physical entities throughout their lifecycle and create real-time connections between the physical and virtual worlds to monitor and control physical objects from any location [25].

Unlocking the full potential of the abovementioned technologies requires new ways of thinking. The centralized architecture is appropriate for some processes [26], but when thinking about the assembly process and personalized products, decentralized structures and appropriate technologies must be used to achieve the highest process performances [27]. In this context, this paper explores the synergistic potential of BPMN and blockchain technology in the management of assembly processes. It explores how the integration of two powerful tools can optimize resource allocation, simplify workflow organization, and mitigate operational risks. Furthermore, real-world applications are shown and emerging challenges are examined, offering insights into how organizations can not only improve efficiency and productivity but also unlock new opportunities for growth and differentiation in an ever-evolving global marketplace.

### 1.2. Human-Centric Approaches

**Figure 2:**
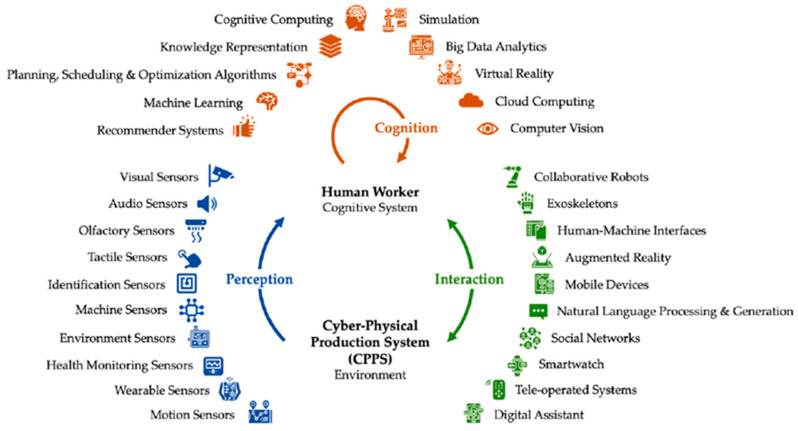
Human–machine symbiosis in Industry 5.0 [33].

One of the central principles of Industry 5.0 is the integration of human-centered approaches into industrial systems. This concept can be understood in at least two complementary ways. The first is the focus on people at assembly workstations. This involves designing the industrial environment to achieve ergonomic and manual work standards that ensure the safety, comfort, and efficiency of workers. This includes the design of the workplace, reducing physical strain, and enabling intuitive interaction with machines. The second direction of human centricity refers to the personalization of products in response to market demands. Industry 5.0 emphasizes agile production systems that are able to quickly adapt to new customer requirements, changing orders, and dynamic production schedules. This enables companies to meet consumer expectations for personalized products without compromising on efficiency or quality. Closely linked to both directions is the need for advanced human–machine interaction. As humans and intelligent systems increasingly work together in manufacturing, new human–machine interfaces that combine both hardware and software elements are required. These elements represent a powerful tool in advancements in industrial personalization, which are especially helpful in the management of assembly processes. It is known that humans’ role in the past was very important and that the assembly of personalized products relied on human skills. Skilled workers played a crucial role in making precise adjustments, performing complex tasks, ensuring consistency in quality, and making decisions [28]. Technological improvement has transformed the human role in production and assembly processes from being work intensive to knowledge intensive [29]. Thus, people still have much to do in industrial processes because they have to be included in generating innovations and in managing and optimization processes. This paper emphasizes the human-centric approach, which is crucial to successfully implement new technology such as digital twins. Traditional digital twins are focused on the replication of real physical objects, products, and systems. The main purpose of this concept was to define, verify, and validate a product or system with high fidelity, including all its characteristic behaviors, data, flows, and failure scenarios [25]. Since the traditional model of the factory’s DT does not consider the human role in an appropriate way [30], efforts have been made to extend the traditional DT to consider the impact of collaborative intelligence interactions [31]. Moreover, the researchers propose a collaborative intelligence interaction framework called Human-CENTRO, also considering that a DT cannot always be fully autonomous and can require human intervention; the framework also assesses how a DT’s architecture can be complemented and work with humans. One direction of their research involves humans assisting machines, where the goals are to train machines to perform specific tasks, explain the results of those tasks, and sustain the responsible use of machines. The second direction involves machines assisting humans, where the goals are for machines to amplify human cognitive strengths and physical capabilities, interact with customers and employees, and embody human skills to extend their physical capabilities. Generally, adapting technology to humans must coincide with training people how to use new technologies [32]. Human centricity should not be a one-way road. Otherwise, technologies will not unleash their full potential [8]. Reintroducing human workers to the factory floor is mentioned in many studies with a focus on creating a meaningful and sustainable socio-technical system that effectively integrates and supports the use of smart and intelligent technologies within organizations (Figure 2).

To create human-centered digital twins (HCDTs), HCDT-driven CPSs and sensors can be deployed to collect digital data from humans; these tools are further integrated with different modeling and simulation tools to support the whole DT framework, which is crucial for this research. Many mature solutions have been developed to collect data from physical systems, such as collecting data from cobots or other devices in the production process [34]. However, the practice of collecting data from humans is still in progress. Various human motion tracking sensors developed in recent decades are able to provide accurate results [35]. When designing the human-centric environment, there are areas to be aware of. Based on the application area of a human-centric digital twin, the selection of sensors and tools for system integration can be revised according to the physical environment (such as sensors for air quality and flow, light, and noise), needs for human–robot collaboration (cameras, force sensors, and IR sensors) [36], measurement of vitals (biological sensors), and human digital twin inputs (can be direct or indirect, such as voice commands, face recognition, and visual or motion sensors) [37]. This can ensure an appropriate environment for people.

### 1.3. Digital Twin

When starting to use a digital twin, it is necessary to understand the difference between digitization, digitalization, and digital transformation. Digitization is the process of converting analog data into digital form. This is essential because digital data is the only form acceptable to industrial information systems. This enables the creation of business value for processes that require data. Furthermore, the term digitalization is used for the adoption of information technology in organizations. Moreover, it can be defined as the strategic use of digital data to improve production processes. Finally, digital transformation is the application of technology and software to build new models, processes, and systems that should enable greater competitive advantage and higher efficiency within the system.

The digital twin concept dates back to 2002, when the idea of a virtual space containing the information of and linked to a real space emerged in the field of study of complex systems. Recent definitions of digital twin can be found in various sectors, with a wide use and diffusion of the concept of a virtual replica of physical entities whose purpose is to manage, optimize, and control the physical asset itself. In the manufacturing sector, the idea of the connection between physical components and virtual models is widened, adding the necessary flow of data between the physical asset and its virtual part in order to monitor the actual object in real time, supporting simulations, analytics, and control capabilities of the dynamic virtual model.

The digital twin of a facility, through which relevant data in a database is used with a backflow of information for decision-making, will continuously be a live facility management tool for daily review of the facility’s expected behavior and performance.

The main aims are

- The optimization of internal processes;
- The development of new business models;
- Innovations in the organization;
- Improving communication with customers;
- The formation of new communication networks.

**Figure 3:**
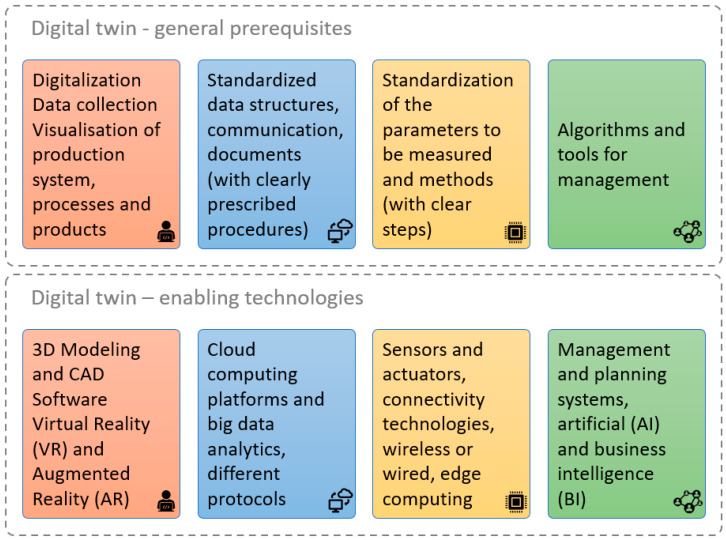
General prerequisites and enabling technologies for digital twin formation and usage.

The general prerequisites, Figure 3, start with the ability to capture relevant data from machines, sensors, and processes, followed by the digitalization and visualization of production systems, workplaces, processes, and products. These elements ensure that the physical environment is reflected in the digital space. Another prerequisite is the availability of human–machine interfaces (HMIs) that enable the operator to interact intuitively with the digital representation and influence its behavior. In addition, properly documented standards, procedures, and protocols are required to ensure that the digital twin accurately and consistently reflects the real process. Finally, sophisticated algorithms and computer tools are required to interpret data, optimize processes, and efficiently manage the digital twin’s environment to support informed decisions in the real system.

Enabling technologies include 3D modeling and CAD software, virtual and augmented reality tools, cloud computing platforms, big data analytics, and sensor networks. In addition, effective digital twins require the use of standardized communication protocols, common data structures, and reliable methods for data transfer. These standards are essential to ensure interoperability and consistency and secure integration across heterogeneous devices and platforms. Together with advanced connectivity technologies, AI-driven management systems, and business intelligence applications, these enabling technologies allow digital twins to operate in real time and deliver actionable insights across the entire production lifecycle.

### 1.4. Challenges and Application of Blockchain

The assembly process is characterized by multi-faceted and distributed operations; thus, the ability to manage and harmonize these diverse elements effectively represents the challenge. Blockchain technology is a mixture of well-known “smart” technologies, first introduced by Satoshi Nakamoto in 2008, as the initiator of the digital currency known as Bitcoin [38]. The main requirement in blockchain is trust. In addition, these features are obtained by using a distributed peer-to-peer network based on open-source software running in each node, each containing a copy of the transaction database, known as the blockchain. Blockchain is a peer-to-peer distributed ledger of transactions stored in a chain of blocks that permits records to be shared with all the network nodes without a central authority. Blockchain typically includes the following capabilities, which may be dependent on the platform used [39]:

- Shared ledger: A data structure that is distributed locally and shared between different participants;
- Permissioning: Secure and authenticated transactions that ensure privacy and transparency of data;
- Consensus: Transactions are endorsed by relevant users that ensure immutability and traceability of data.

Transactions on a blockchain permit any data (for example, when it is applied in assembly process management, including the state and location of products and materials) or digital assets to be applied to some state and values and saved as an address on the blockchain. Each transaction in the blockchain must be verified by a majority of the nodes in the system before being appended to the blockchain. The nodes must agree, and only then can the transaction be appended. This process is known as consensus [40]. A large number of nodes and economic incentives guarantee the survival of the network and its robustness; equality and openness of all nodes make the network decentralized, the transparency and immutability of the blockchain enable trust, and the consensus mechanism used to add new transactions to the blockchain (proof of work) guarantees security against massive attacks. This is why a new trend has appeared where market leaders in the manufacturing sector are trying to use the inherent features of blockchain to improve fields that have previously been an obstacle to technical development.

Table 1 compares the main areas in which blockchain offers advantages over existing technologies that characterize Industry 4.0/5.0 applications and illustrates the improvements that can be achieved through its implementation. Blockchain is transforming manufacturing, and there are some vivid examples from different industries. Blockchain can help manufacturers track each of these parts before, during, and after production. For example, car manufacturers build cars from many thousands of components from hundreds of sources. Ultimately, they are responsible for the reliability and safety of the vehicle, but if a problem arises that requires a product recall, they need to find and replace the parts that are causing the problem. The industry’s private blockchain can help manufacturers track information about components from all suppliers. As suppliers manufacture and deliver components, they enter the data as blockchain transactions. As the components are tested and then installed, new details are added. In the event of a problem, the manufacturer can immediately determine where the problem part came from, which products it is installed in, and which route the component has taken in the production cycle. This allows the manufacturer to track and solve problems much more quickly and efficiently. The complexity of the sector is further increased by emerging new trends. Fortunately, with blockchain, manufacturers have a powerful new tool at their disposal that can help them reduce costs, optimize processes, improve supply chain management, and increase accountability. To further support the application of blockchain technology, this paper provides an example of how to apply it at the process level for assembly purposes.

Since blockchain technology enables the secure exchange of data in a distributed manner, it can affect the configuration of relationships and the sharing of information between participants. For example, an assembly supply chain is fundamentally different from an ordinary supply chain. Material exchanges between upstream and downstream supply chains are not finished products but key components or semi-finished products. At the same time, suppliers of basic components and assemblers (manufacturers) can jointly or directly influence consumer preferences. With the use of blockchain, consumer preferences, pricing, and key quality decisions will change, and new conflicts will emerge among members of the supply chain. In this context, it is essential to understand the impact on the assembly supply chain if some suppliers of key components decide to attack the market with imitation products. Similarly, before adopting blockchain technology, it is necessary to fully understand the impacts of blockchain application on the assembly supply chain [41]. A wide range of blockchain-based traceability systems have been proposed. Early concepts highlighted the technology’s suitability for enabling greater supply chain transparency and data integrity. The first models for managing digital agents and blockchain-based data inputs in supply chain units were created. Smart contracts are increasingly used to represent traceability functions, such as receiving or transferring products, which has enabled a closer connection between physical processes and virtual transactions. Later, the focus on the digital display of physical assets was further strengthened, which can be considered a starting point for the traceability of production processes. At the same time, the idea of combining off-chain data storage also emerged, enabling performance improvements by focusing on application-relevant data within the blockchain network. In this sense, concepts for cooperation in smart manufacturing services, lifecycle data, and digital twins enabled by blockchain have been developed. According to Garcia et al. [42], there are relevant technical characteristics derived from a review of the existing literature such as a blockchain platform or architecture, process modeling languages, smart contract technologies, and a process execution platform or process engine. From previous analysis, it can be concluded that blockchain does not necessarily replace these technologies but complements them by closing their critical gaps. All mentioned studies point to the further need to detect the possibilities of the mentioned topic and how to integrate the benefits of new technologies. While ERP, MES, SCADA, PLM, RFID, and digital twin technologies already provide comprehensive support for process control, monitoring, and optimization in the sense of Industry 4.0 and 5.0, their scope of application is largely limited to internal company use or trusted partnerships. These systems may be effective for automation and interoperability but remain centralized, vulnerable to tampering, and dependent on trust in a single system owner. This is particularly important in the context of personalized production, where multiple suppliers, partners, and service providers can be involved in each product configuration. Trust must extend throughout the supply chain to ensure that customer-specific requirements, design data, material flows, and quality information remain transparent, tamper-proof, and verifiable at every stage. Blockchain enables this level of distributed trust, which is a prerequisite for scaling personalized production beyond individual companies.

### 1.5. BPMN Standard

At the beginning, business processes were mostly recorded textually, but as the processes became more and more complicated, they included various participants, details and exceptions, and the need for a more precise description arose. Existing approaches in smart manufacturing and assembly are traditionally supported by the automation pyramid, which integrates different levels of control and management systems. At the field level, sensors and actuators capture process data, which is then processed by PLC and SCADA systems for control and monitoring. The MES coordinates production activities, while ERP and PLM systems enable higher-level planning, resource allocation, and product data management. Together with IoT, these technologies enable structured process control, optimization, and traceability. Moreover, these technologies facilitate decentralization in the context of data exchange. Therefore, when approaching business optimization and redesign tasks, a full and precise understanding of the existing process is crucial. The graphical display enabled the construction of a systematic holistic view of processes that can be easily read and understood by all those involved, such as process owners, managers, technical engineers, and many others, regardless of their level of technical understanding and expertise. There are various existing techniques and languages for graphical modeling of processes and their efficiency. For this research, the Business Process Model and Notation (BPMN) was applied, consisting of graphic elements and formalized records, and it has the status of a professional norm. The primary goal of BPMN is to provide a notation that is easy to understand for all business users, from business analysts who create initial drafts of processes, to technical developers responsible for implementing the technology that will perform those processes, and ultimately to business people who will manage and monitor the processes. In this context, BPMN is well known to achieve higher productivity, efficiency, and reduced costs. Approved as an industry standard by the Object Management Group (OMG), BPMN provides organizations a systematic framework for modeling and optimizing business processes. Moreover, the versatility of BPMN goes beyond visualization, allowing seamless translation into executable code through languages such as BPEL (Business Process Execution Language), thus bridging the gap between process design and implementation. The integration of BPMN, BPEL, and blockchain technology appears as a compelling solution, promising a revolution in the assembly process management. By combining the advantages of BPMN in the modeling and optimization of business processes with the immutability and decentralization of the blockchain, greater transparency, security, and data integrity are enabled in every step of the assembly process. This synergy not only improves efficiency and reduces the risk of errors but also creates an environment of trust among all participants in the process. The assembly process is extremely complex and specific because of its decentralized nature, usually requiring coordination on multiple locations. This dispersion demands significant effort regarding management. This process itself involves many actions regulated by a variety of principles and laws, always including technological, economic, sociological, and ecological aspects. Fundamental determinants of assembly are the geometric and physical properties of components, product structure, and order fluctuations. By recording every event in a distributed ledger protected from unauthorized changes, blockchain reduces the risk of fraud, eliminates middlemen, and enables the real-time tracking of goods and components in the whole chain of participants. This not only improves traceability and accountability but provides trust among stakeholders, fostering a collaborative system that leads to growth. As mentioned in the explanation of Table 1, the distributed and interoperable character of Industry 4.0 technologies provides a natural starting point for blockchain integration. The immutability and decentralized nature of the blockchain ensure that every event in the assembly process, whether material flow, product quality, or decision-making, is securely recorded and can be checked by everyone involved. This integration creates an environment of trust, improves collaboration in distributed networks, and strengthens the transparency and accountability of complex assembly processes.

## 2. Methodology

To conduct this research on a real example, i.e., to integrate BPMN, digital twin, and blockchain technology, a three-step process was used. In the beginning, it was necessary to prepare and standardize all documents according to the explanations shown in Figure 3. Furthermore, advanced technologies were combined to prepare the digital twin. Three-dimensional modeling tools help to create detailed replicas of physical objects. Cloud computing provides the necessary computational power and storage for handling amounts of data collected from sensors and generated by the digital twin. Sensors and actuators provide real-time data from physical systems. Connectivity technologies facilitate communication between physical objects and the digital twin. A comprehensive understanding of the characteristics of IoT will help to better apply it to the manufacturing sector and provide new opportunities regarding scalability, heterogeneity, interoperability, autonomy, security, and openness [2].

To define the blockchain features within the process model, BPMN was used in this case. This could be justified because BPMN is an international standard for business process modeling and is the most supported notation by many process execution engines, thus facilitating its deployment in real environments [43]. In this case BPMN ensures that all steps are followed systematically, from parts procurement and quality control to component assembly and packaging. BPMN is followed by execution, which is the purpose that BPEL (Business Process Execution Language) serves. BPEL is a technical tool which enables the automatization and execution of these business processes within the IT system. This example shows that BPMN focuses on the visual representation and design of the workflow, where business experts and business process designers’ model and analyze business activities. Once the processes have been modeled using BPMN, the next step is to execute the modeled processes using BPEL. BPMN therefore enables collaboration between business and IT environments, while BPEL uses these models to implement and execute them within the information system. After this phase, we identified the important areas in the assembly process where blockchain technology can add value. In this case the blockchain ensures data validation within the network that supports the digital twin and provides security and immutability of the recorded data. Entities that interact in the BPMN model represent the blockchain nodes, and the tasks in BPMN correspond to transactions executed via smart contracts on the blockchain. Some decision-making key points are not automated but should be checked by a manager or worker before being further executed because this is a human-centric digital twin. Also, some crucial tasks in the assembly process (also in the BPMN process) are validated before being added to the blockchain. This methodology enhances process transparency and traceability as important aspects of the assembly process. The real-time transparency of the entire assembly process ensures that those involved can easily track progress and identify bottlenecks. Production stoppage due to a wrong piece or incorrect assembly is visible, and it can be solved quickly. If a fault is found, it can be traced back to the exact step or component that caused it, which improves root cause analysis.

**Figure 4:**
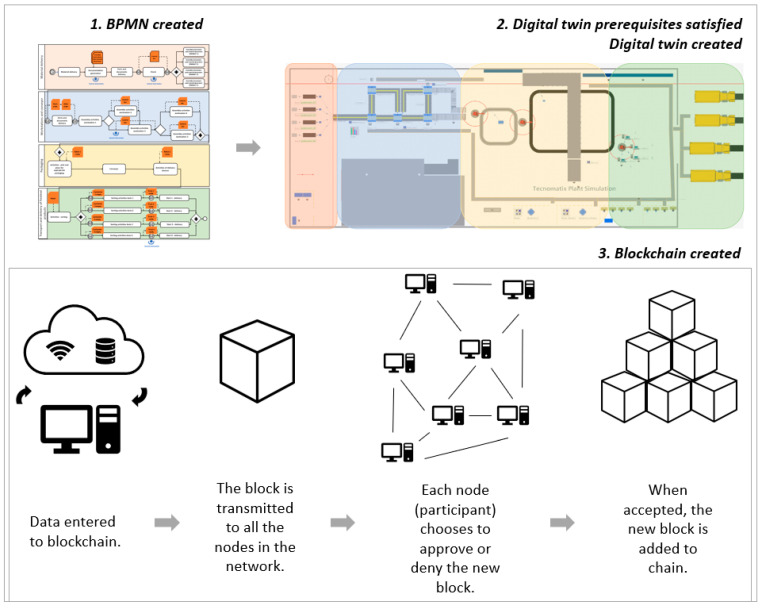
Three-phase designs with integration of BPMN, DT, and BC technology used for real assembly process.

Figure 4 illustrates the three-phase designs with the integration of BPMN, DT, and BC technology used for a real assembly process. Phase 1 begins with the creation of a BPMN diagram. The process is described in such a way that it can be understood by both technical and non-technical operators, ensuring a common reference for optimization and implementation. Once the process model is defined, phase 2 begins. The digital twin provides a dynamic, real-time representation of the physical assembly process and enables monitoring, simulation, and optimization before and during actual execution. Human–machine interfaces support the operator’s interaction with the digital environment and ensure an accurate reproduction of real-life conditions. Phase 3 is related to blockchain creation. Before the block is created, data is gathered with a real-time sensor from a real workstation. The human–machine interaction is supported by an Arduino-based platform equipped with two infrared sensors; the first sensor detects when the material is taken from the box, while the second registers the completion of the assembly task. The time difference between these signals was processed and transmitted to the cloud, where it was integrated into the digital twin for validation and monitoring. The BPMN workflow was directly coordinated with the digital twin and blockchain technologies by mapping BPMN entities to blockchain nodes and BPMN tasks to transactions executed via smart contracts. The decision points defined in the BPMN were synchronized with the event logs of the digital twin. Once validated, these events were recorded in the blockchain to ensure the immutability and traceability of process execution.

## 3. Case Study: The Assembly Process

**Figure 5:**
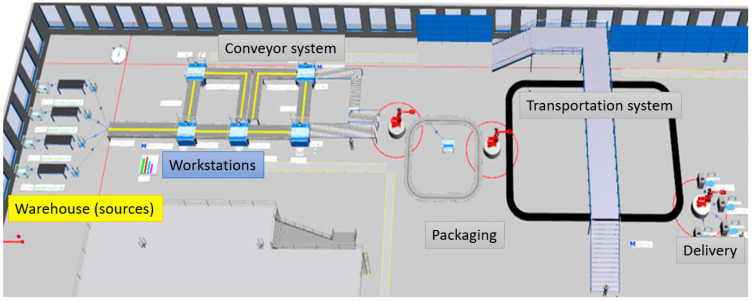
The assembly process in Tecnomatix, Plant simulation 16.

The methodology described in the previous section was tested on a real example in a laboratory environment. Figure 5 shows the entire process, presented in Tecnomatix, Plant simulation 16 (Siemens Digital Industries Software, Plano, TX, USA).

The process starts with warehouses of parts and sorting procedures and then continues with the distribution of parts at specific workstations via a conveyor. There are five workstations for assembly, and when finished, the final products move to the packaging process.

**Figure 6:**
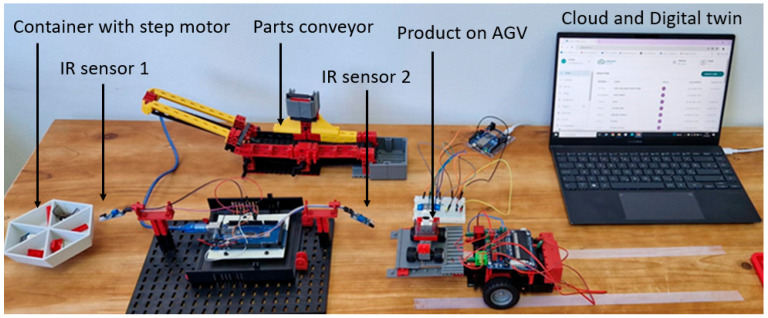
Smart assembly station.

The intelligent workstation was developed as a part of a master thesis [44], and it comprises several elements that enable automation, monitoring, and optimization of the assembly process. Each workstation in the laboratory was equipped with microcontroller Arduino boards, sensors, and actuators connected via Wi-Fi. This powerful combination enabled the creation of a smart workstation. The Arduino boards used their own integrated development environment, where codes prepared according to the algorithms were developed and uploaded to control the process. Arduino cloud platform served to connect Arduino hardware to collect data, monitor and control the process, and interact with Arduino devices (Arduino, Turin, Italy) remotely. Figure 6 shows (from left) a rotating container for assembly components attached to a stepper motor, IR sensors, and an Arduino R4 board for data collection (via Wi-fi). The container for assembly components rotates as the assembly instructions define the steps, so the worker can easily pick up the required component at an exact time. Rotation is made possible by a four-stroke stepper motor whose code is written in C++ based on assembly instructions. After the last rotation of the container, i.e., when the worker removes the last component, the sensor connected to the Arduino platform starts measuring the time and records the duration of each assembly step. The moment the worker assembles the last component, he raises his hand to leave the sub-assembly component, and there is an IR sensor at the right place to properly detect the end and record the time. The Arduino microcontroller plays a key role in data collection and processing. This data is used for efficiency analysis, process optimization, and identification of assembly bottlenecks. Furthermore, it is used to continuously rebalance the assembly line. After the last component has been assembled, the autonomous vehicle picks up the assembled car and transports it to the next workstation. The vehicle follows the lines drawn on the work surface, enabling precise movement along a predefined path. This automation reduces the need to move parts manually, speeds up the process, and reduces the possibility of human error. The conveyor on which the defective parts are placed can also be seen in the background.

**Figure 7:**
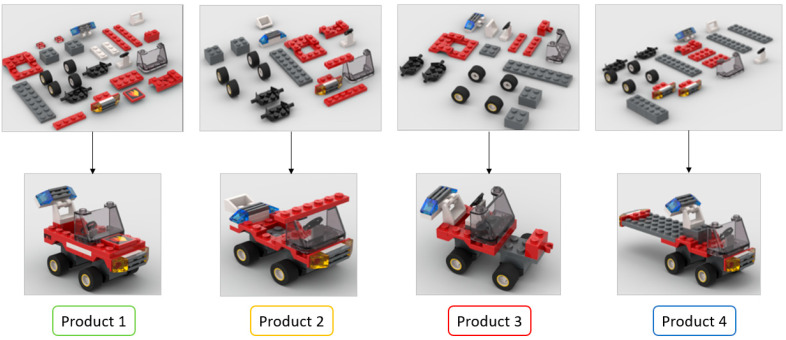
Presentation of products.

This workstation is part of a larger network of decentralized workstations that exchange data with each other using blockchain technology. Each workstation has access to all collected data, which ensures the security and availability of the data in the event of a failure of one of the stations. The data is stored in the blockchain, which prevents its loss or unauthorized modification. Four different products were assembled within the same assembly process to test the system’s performances. Four products were used with different configurations of parts, ready for further assembly and testing. The bill of materials according to assembly standards was used in this case study, and each part has an identification number for Products 1, 2, 3, and 4. Figure 7 shows four different products assembled using the same assembly process.

The positional weighted method is an assembly line balancing technique used in this case to assign tasks to workstations in such a way that the line operates efficiently, minimizing idle time and balancing workload across stations. Input data for this method is shown in Table 2.

Sensors added to workstations collected data regarding the time taken to complete assembly tasks. Infrared (IR) sensors which work with the Arduino platform were used to collect data for each element of work. IR sensors emit infrared light and detect the reflection of that light from objects (the time starts when the worker takes each part to perform assembly, and when assembly is finished for a semi-finished part, the time ends, because the sensors were positioned to track the movement of the worker’s hands), as shown in Table 3.

**Figure 8:**
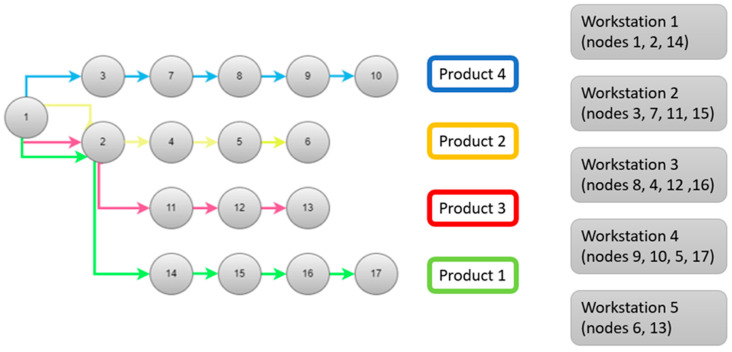
Precedence diagram with elements of work presented as nodes.

These data were used to define a better layout for the process; it was another input for the algorithm based on positional weight method. According to the results, a precedence diagram was generated, as shown in Figure 8. Takt time was calculated, and the minimum number of workstations was defined (5 workstations). Elements of work were assigned to workstations to achieve the appropriate workload.

**Figure 9:**
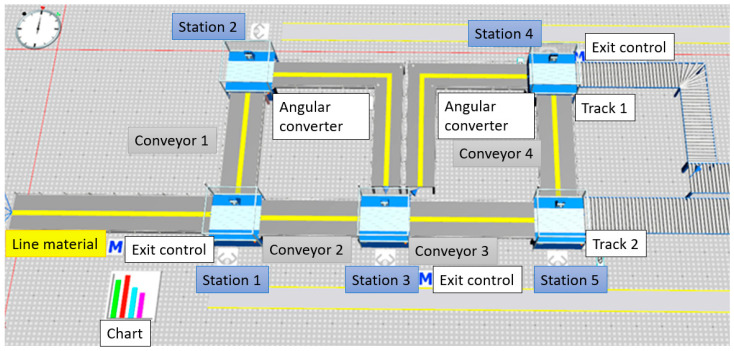
Digital twin in Tecnomatix plant simulation software.

Digital twin was created in Tecnomatix plant simulation software, as shown in Figure 9. Digital twin collected mentioned data and gave the results for optimization of material handling, logistics, workstation utilization, and work requirements. This allows quick bottleneck checks, confirmation of transported material, and review of resource utilization over time for multiple alternative processes. By using stochastic tools with object-oriented 3D modeling capabilities, the accuracy and efficiency of production and overall system performance are increased.

**Figure 10:**
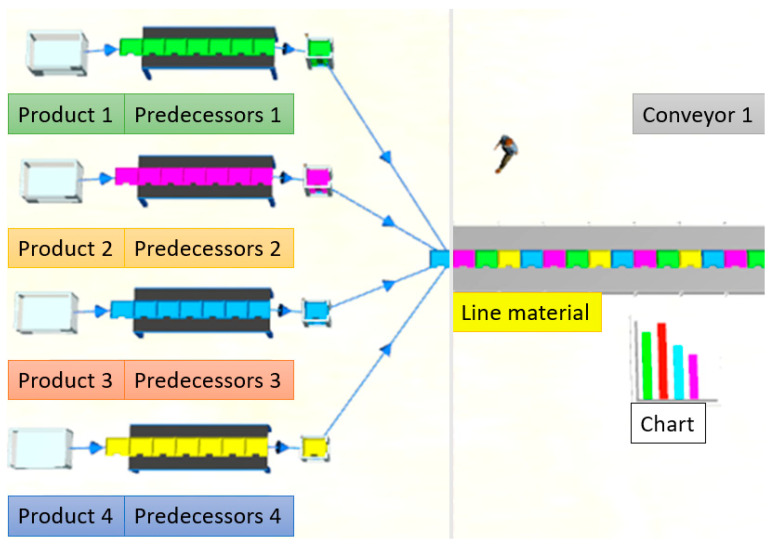
Sources (warehouses).

Before the common conveyor called line material, a model was created showing four different sources sorting the material depending on which product will be built. Sources are colored differently for easier differentiation further in the process, as shown in Figure 10. Given that each car is not subject to the same workstations, it was necessary to create an output method of material movement for individual stations, which is programmed within simulation software.

**Figure 11:**
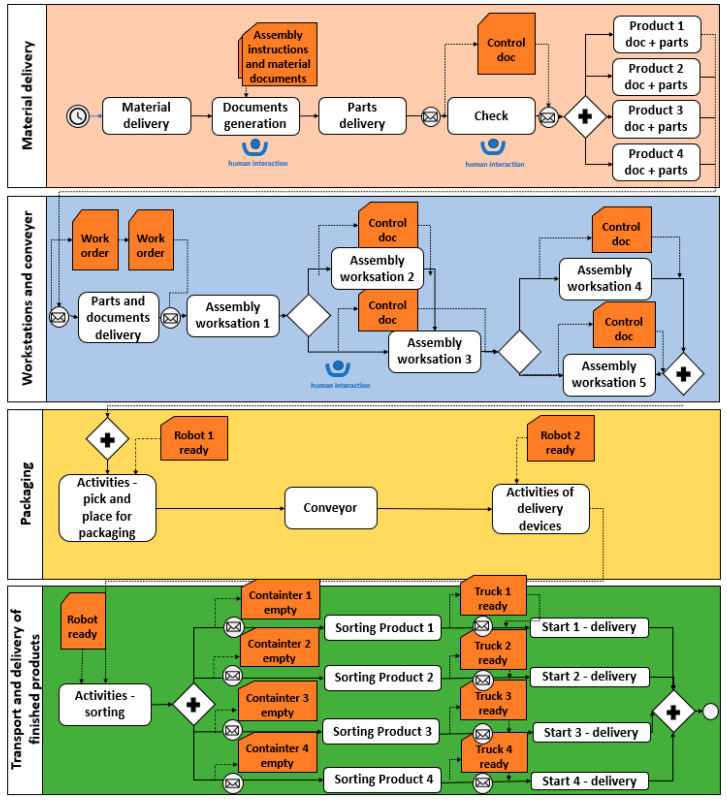
BPM of assembly process to define blockchain features.

To ensure appropriate data collection and human interaction points, in the background, BPM was created for this assembly process, as shown in Figure 11. This BPM representation is used to further model BC features. This enables the process execution and maintenance of process workflows. It allows for data collection and communication between different objects within the process. As a human-centric digital twin is tied to BPMN, it is shown where in the process a human interacts with a digital twin to monitor the process and make decisions regarding the results.

According to [25], the taxonomy of the digital twin is used to represent the human roles vs. dimensions within the human-centric digital twin for the case study, as shown in Table 4. Key rows in this table refer to how data is created, connected, and shared. Data link refers to how data is transmitted from various sources, such as sensors or historical data. Purpose refers to goals for each role in the human-centric digital twin. Accuracy level is presented for each role. Synchronization describes how the data from the digital twin is synchronized with real-time processes. Data input lists the type of data entered into the system for each role. Time of creation is important to emphasize because it specifies the moment the data is generated and ready to use, where real time is crucial. The main advantages of each role are highlighted. Further needs represent the guidelines to realize in future.

This research reveals key benefit areas visible in the presented case study within a system where human factors are integrated with digital technologies:

- Enhanced worker efficiency with reduced mental and physical workload;
- Improved planning and process optimization;
- Real-time decision support.

## 4. Results and Discussion

Tecnomatix plant simulation was chosen for its discrete event simulation capabilities, which make it ideal for analyzing complex manufacturing systems. This tool enables precise statistical analysis of key aspects of the production process, including optimization of material handling, logistics, machine utilization, and labor requirements. The simulation included analyzing the cycle time at different work stations, identifying potential bottlenecks and ensuring that each station was evenly utilized. Another objective was to investigate possible system improvements and provide the benefits of human interaction. The simulation provides an overview to identify improvement opportunities, such as improvements in transportation, additional automation, or optimization of resource usage. The program offers stochastic tools that enable the simulation of different scenarios and a risk assessment. In addition, object-oriented 3D modeling capabilities allow for real-time visualization of the assembly process, which increases simulation accuracy and enables more precise analysis. After the simulation, the general statistics of each workstation are displayed in Table 5. There are many other parameters visible, such as average down time per event or standard deviation of downtime. These indicators are essential for identifying potential inefficiencies and ensuring optimal system performance. For each workstation, additional critical parameters were monitored, such as waiting times, downtime, failure times, and throughput.

Furthermore, when analyzing a specific product, there are visible important parameters such as waiting times, downtime, failure times, and throughput. The results of the digital twin show that the system throughput is balanced across all outflows at around 452 parts per day. However, a more detailed breakdown shows that transport (53–59%) and warehouse waiting times (33–38%) dominate resource utilization, while direct production tasks account for less than 4%. These insights are made possible by real-time data collection using infrared sensors connected to an Arduino platform, with data being transferred to the cloud via standardized protocols for processing and storage. The cloud infrastructure ensures scalability and interoperability so that the digital twin can continuously reflect the current state of the assembly line. BPMN models applied to this data layer serve as the structure and formalize the workflow logic, ensuring that each step (from warehouse to packaging) is executed according to the defined process rules. Blockchain technology is behind to secure workflow, by converting BPMN tasks into verifiable transactions that are executed via smart contracts, while ensuring that all collected data and decision points are immutable, verifiable, and transparently shared (Table 6). This table shows how a digital twin goes beyond simple throughput measurement. Traditional monitoring could only show that all drains produce equally (452 products per day). However, the digital twin reveals where inefficiencies occur (i.e., transport and storage) and provides actionable insights for line balancing, scheduling, and resource allocation. By applying optimization methods such as the positional weight method, these waiting and processing times can be reduced, resulting in greater efficiency without the need to increase physical resources.

Besides these results, there is a downtime reduction metric considered within the presented model. This metric is specific because it is related to human interaction within the system and is used to capture the impact of human interaction to solve problems regarding system downtime, such as transportation. This highlights the important role of human operators in assembly systems, maintaining efficiency and performance. Through this case study, it is proven how human reaction is faster than automated response as humans can identify transportation problems faster, responding to alerts and adapting to workflow in real time. This *downtime reduction metric* is defined as follows:

(1) $$downtime\;reduction\;metric=\frac{downtime\;without\;intervention-downtime\;with\;intervetnion}{downtime\;without\;intervetnion}$$

The metric is expressed as a percentage. By incorporating human intervention, the *downtime* was reduced. According to the results above, workstations experienced about 52% of *downtime*, which represents a problem. Human interaction helped to reduce this to 40% in the first assembly iteration, and this metric demonstrated the benefits of human centricity that could not be solved within this case study in real time.

(2) $$downtime\;reduction\;metric=\frac{52-40}{52}=0.23=23\%$$

When seeing an alert (LED light) about exceeded time for transportation, the operator presses the button to solve the issue. After the intervention, the end time is recorded via IR sensors and the *downtime* metric is calculated. Furthermore, the total number of interventions over time is counted within code. The setup from this case study provides quantitative data on human interactions, enabling better flows within the system based on the transport problem method.

In this research, it is shown how the general organizational sustainability concept is realized through the usage of key technologies aligned with human-centric approaches. A new prototype of digital twin that optimizes an assembly system based on algorithms and humanoid decision-making is provided as a proof of concept. The human-centric digital twin for industrial application is represented through a case of personalized products. The proof of concept is demonstrated by solving the case of the assembly process for different products. Comparing this research with reference [45], it is confirmed how several critical relationships between key model variables, particularly regarding the influence of technological readiness, affect digitalization and product personalization. Moreover, the findings in this paper emphasize the critical role of human centricity, prerequisites for digital twin, BPMN, and blockchain technology integration in successfully implementing digitalization. For organizations aiming to enhance their product personalization capabilities, it is not enough to be technologically ready, but they have to ensure that their internal operations are well-integrated and aligned. Also, in [45], the results showed that Industry 4.0 technologies and digitalization positively affect product personalization if they support enterprises’ internal and external integration and value chain.

## 5. Conclusions

This research examined the challenges facing modern manufacturing companies in the context of turbulent market conditions and rapidly evolving technologies. To remain competitive and adaptable, companies have to take advantage of all the benefits that the new era offers. In this research, the combination of digital twin, cloud connectivity, BPMN, and blockchain creates a holistic system that not only identifies bottlenecks in real time but also ensures trust, accountability, and collaboration across the entire assembly network. The virtual replica of the real assembly process (digital twin) makes it possible to monitor the condition of the real object in real time and to simulate different situations using accurate and updated data. In this context, the integration of the business process model and notation with blockchain technology has proven to be a promising solution for optimizing the management of the assembly process.

The main contribution of this research is the framework that combines digital twins, BPMN, and blockchain technologies in a unified system to optimize assembly processes. Digital twins provided quantitative insights into bottlenecks and showed that transport consumed up to 59% and warehousing consumed up to 38% of the total resource time, while production tasks accounted for less than 4%. These findings highlight a critical imbalance in resource allocation, where supporting activities such as transport and warehousing dominate the total process time, while the core value-adding tasks of production account for only a small proportion. This information is crucial as it clearly shows where inefficiencies lie and provides a solid basis for discussing targeted improvements. By reducing unnecessary transport, optimizing warehouse processes or even redesigning the layout and material flows, overall efficiency can be significantly increased. In this way, the digital twin not only quantifies the bottlenecks but also identifies specific opportunities for redesigning processes and reallocating resources that can reduce or even eliminate these inefficiencies. In addition, BPMN provided a structured model of the assembly workflow, while blockchain ensured that every execution was securely recorded, immutable, and transparently accessible.

Finally, it is important to note that while Industry 4.0 has brought significant technological advances, it often neglects two important aspects—human centricity and sustainability. In response to this shortcoming, Industry 5.0 was developed to complement and extend the scope of Industry 4.0. Industry 5.0 represents a significant transition in the industrial sector, combining technological advancement with a focus on people and redefining the dynamics of work by emphasizing social sustainability and sustainable development. It recognizes the need for equitable and comprehensive workforce training that prioritizes worker wellbeing alongside technological adaptation. The rapid pace of technological advancement requires significant investment in skills development, emphasizing the importance of continuous learning and training and the acquisition of new skills to enable the workforce to meet new challenges. In this context, Industry 5.0 advocates inclusive training programs that meet the diverse needs of the workforce while addressing the challenges of and seizing the opportunities for social and environmental sustainability. This transition aims to create a sustainable and inclusive industrial future where technological progress, human values, and environmental concerns are harmonized. Industry 5.0 sets a new standard for industrial operations and professional development and positions itself as a milestone for modern, responsible, and sustainable industrial practices to ensure that the future of industry is at the service of people and the environment. For future research, the proposed methodology should be applied o additional case studies, including the real industrial case study. Furthermore, the ExCurS project should incorporate methodologies and tools other than digital twins, which will be further investigated to supplement the research already performed during the project.

## Figures and Tables

**Table 1:** Important areas for blockchain implementation in the management of assembly processes and products.

Area	Possible Advantages Over Existing Technologies
Traceability	While RFID (Radio-Frequency Identification) and the MES (Manufacturing Execution System) already make it possible to track components, blockchain ensures unalterable and tamper-proof records of every component and every raw material, which can be viewed by multiple parties in real time.
Process optimization	The existing ERP (Enterprise Resource Planning)/MES (Manufacturing Execution System) automates inventory and planning. Blockchain complements this by enabling smart contracts for cross-company automation (e.g., automatic reordering of parts with supplier verification), reducing delays and disputes.
Workforce management	Existing systems log work activities internally. Blockchain can add secure and auditable personnel records (hours, tasks, and equipment usage) that can be transparently shared with contractors or partners to ensure confidence in payroll and compliance reporting.
Inventory management	ERP/MES optimizes warehouse management, but blockchain can add tamper-proof records of material flows across company boundaries and reduces fraud or tampering risks in supplier interactions.
Quality control	Quality control (QC) reports are usually processed within MES/PLM (Product Lifecycle Management). Blockchain provides secure audit trails and enables the trusted exchange of QC data with suppliers, customers, and regulators and supports compliance with standards.
Cost management	Currently, cost monitoring is centralized. Blockchain provides transparent and verifiable cost records across multiple partners, reducing conflicts in joint assembly projects.
Protection of intellectual property	Blockchain uniquely enables secure timestamping and proof of ownership for designs, algorithms, and process data, ensuring secure innovation management that goes beyond what PLM offers. This is a crucial point for workers because a safe environment is enabled to support creativity and innovation, which will be recorded, safe, used, and adequately rewarded.
Data sharing	This includes sharing all information with the stakeholders involved in the assembly process to facilitate communication and ensure the accuracy and safety of data. Blockchain technology enables the recording of verified and immutable data, thereby reducing the risk of data manipulation and conflicts of interest.
Customer involvement	Customers can selectively gain access to verified assembly data, increasing transparency, trust, and satisfaction beyond what current IT systems allow.
Product lifecycle management	PLM systems capture lifecycle data, but blockchain secures the entire immutable lifecycle progression (from design to after sales) and supports sustainability tracking and regulatory compliance.

**Table 2:** Elements of assembly work (assembly step breakdown).

Element of Work (Assembly Task)	Assembly Module
1	Basic module assembly wheels (4 pcs), gray cube 2 × 2, plate 2 × 8
2	Module + wide red wheel plate
3	Module + narrow red wheel plate
4	Narrow red wheel plate, steering wheel, plate 1 × 4
5	Visor, front bumper
6	Light holder, rear blue lights
7	Plate 2 × 4, steering wheel
8	Visor, front bumper 2
9	Narrow red wheel plate, cube (gray) 2 × 2, light holder, rear blue lights
10	Rear yellow lights
11	Cube 2 × 2, steering wheel, plate 1 × 4
12	Visor, cube with hook
13	Plate 1 × 4, light bracket, rear blue lights
14	Plate 1 × 4, narrow red wheel plate
15	Cube 2 × 2, plate white 1 × 4, steering wheel, plate 1 × 6 (2 pcs)
16	Front bumper, plate 2 × 4, mark, visor
17	Rear red lights, cube with hook, light holder, rear blue lights

**Table 3:** Collected data.

*i*	1	2	3	4	5	6	7	8	9	10	11	12	13	14	15	16	17
***t* [s]**	25	3	3	9	12	7	8	9	7	13	10	7	13	8	19	14	14

**Table 4:** Human roles vs. dimensions within human-centric digital twin for assembly process.

Roles	Worker	Planner	Decision Maker
**Data link**	Real-time sensor data from workstations and machines	Historical and real-time data from assembly processes	Aggregated data from all assembly processes
**Purpose**	Optimize workstation and reduce physical workload	Optimize process planning; reduce time, bottlenecks, and warehouse	Data driven decision-making, not relying just on experience, faster and strategic decision-making process
**Accuracy**	High (based on sensor feedback)	Moderate to high	High
**Synchronization**	Synchronized with operations	Synchronization with assembly data	Real-time synchronization across all operations
**Data Input**	Sensors, workstation data	Data from workflows, assembly process, sensor data	Data from workflows, assembly process, sensor data + decision-making algorithms
**Time of** **creation**	Real time during assembly	During process planning	During decision-making phase
**Benefits**	Reduced physical workload, optimized workstation	Reduced workload, higher assembly performances	Faster information flow and prediction, higher assembly performances
**Further needs**	Assistive tools, such as AI-based guidance systems	AI-driven adaptive interfaces, improved data analytics	Adaptive processes, more precise predictive analytics

**Table 5:** Results for monitored workstations (proportions of time working, waiting, blocked, or failed).

Object	Working	Waiting	Blocked	Failed	Portion
Station 1	75.41%	0.99%	10.55%	13.05%	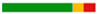
Station 2	53.42%	0.58%	36.50%	9.51%	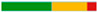
Station 3	72.27%	15.11%	0.00%	12.62%	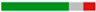
Station 4	81.70%	1.26%	3.97%	13.07%	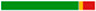
Station 5	20.95%	75.40%	0.00%	3.65%	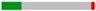

Legend for portion (green—working; yellow—blocked; red—failed; gray—waiting).

**Table 6:** Results for monitored products (monitored parameters for production, waiting transport, storage, lifetime, exit time, and throughput—detailed statistics of the part types which the drain deleted).

Product (Drain)	Production Utilization—Working (%)	Waiting (%)	Transport Utilization (%)	Storage Waiting (%)	Lifetime (Mean)	Exit Time (Mean)	Throughput (Per Day)
Product 1	3.42%	4.54%	58.71%	32.76%	1:15:29	3:09	452
Product 2	3.58%	4.66%	53.53%	37.63%	1:05:45	3:08	452
Product 3	2.91%	4.80%	58.45%	33.36%	1:14:12	3:09	452
Product 4	3.48%	4.83%	57.77%	33.30%	1:14:24	3:09	452

## Data Availability

The datasets used and analyzed in the current study are available from the corresponding author upon reasonable request.
